# Comparative metagenomics reveals taxonomically idiosyncratic yet functionally congruent communities in periodontitis

**DOI:** 10.1038/srep38993

**Published:** 2016-12-19

**Authors:** Shareef M. Dabdoub, Sukirth M. Ganesan, Purnima S. Kumar

**Affiliations:** 1 Division of Periodontology, College of Dentistry, The Ohio State University, Columbus, Ohio, USA.

## Abstract

The phylogenetic characteristics of microbial communities associated with periodontitis have been well studied, however, little is known about the functional endowments of this ecosystem. The present study examined 73 microbial assemblages from 25 individuals with generalized chronic periodontitis and 25 periodontally healthy individuals using whole genome shotgun sequencing. Core metabolic networks were computed from taxa and genes identified in at least 80% of individuals in each group. 50% of genes and species identified in health formed part of the core microbiome, while the disease-associated core microbiome contained 33% of genes and only 1% of taxa. Clinically healthy sites in individuals with periodontitis were more aligned with sites with disease than with health. 68% of the health-associated metagenome was dedicated to energy utilization through oxidative pathways, while in disease; fermentation and methanogenesis were predominant energy transfer mechanisms. Expanded functionality was observed in periodontitis, with unique- or over-representation of genes encoding for fermentation, antibiotic resistance, detoxification stress, adhesion, invasion and intracellular resistance, proteolysis, quorum sensing, Type III/IV secretion systems, phages and toxins in the disease-associated core microbiome. However, different species or consortia contributed to these functions in each individual. Several genes, but not species, demonstrated robust discriminating power between health and disease.

Periodontitis, a microbially induced disease that destroys the structures anchoring the tooth to the jawbone, is the sixth most prevalent disease in the world, affecting over 700 million adults worldwide^1^. The consequences of untreated disease are tooth loss, poor nutritional status, loss of speech and masticatory function. With the annual cost of periodontal treatment exceeding 15 billion dollars in the USA alone, this disease poses a significant health burden^2^. Additionally, emerging evidence implicates periodontitis in the pathogenic pathways of several systemic diseases^3^,^4^, and therefore, the consequences of untreated periodontitis may extend beyond the oral cavity.

Periodontitis is a site-specific disease, with the disease affecting many, but not all teeth in an individual with disease. Also, disease progression occurs through recurrent bursts of destruction followed by varying periods of quiescence. This temporally and spatially haphazard mode of disease progression has been named the random burst model^5^. Several reasons have been proposed to explain the random burst hypothesis, including the quality of plaque, presence of plaque retentive factors and local inflammatory response^6^, however, the mechanism underlying this arbitrariness is poorly understood; and poses a barrier to effective clinical treatment and disease prevention.

An ecological shift in the indigenous microbiome towards dysbiosis is known to play a primary role in the etiology of this disease. While the taxonomic profiles of eubiotic and dysbiotic periodontal communities are well-studied^7^,^8^,^9^,^10^,^11^,^12^,^13^,^14^,^15^, little is known about their genome signatures, and whether community level functional alterations accompany compositional shifts in the transition to and in the state of disease. Even less is known about the selection pressures exerted by a disease-associated environment on the pre-existing microbiome. Also, although several lines of evidence have suggested a role for viruses, archaea and fungi in disease etiology^16^,^17^,^18^, their contributions to the functionality of the periodontal ecosystem has never been explored.

The primary purpose of the present investigation, therefore, was to better understand the functional potentials encoded within health-compatible and disease-associated periodontal microbiomes, using a comprehensive metagenomic approach and computational bioinformatics to characterize correlations between the mycobiome, virome, archaeome and bacteriome. A secondary aim was to investigate if periodontitis is the result of a site-specific dysbiosis or a global shift in the subgingival microbial ecosystem.

## Methods

### Subject and site selection

Approval for this study was obtained from the Office of Responsible Research Practices at The Ohio State University and the study was conducted in accordance with the approved guidelines. Twenty-five periodontally healthy never-smokers (attachment loss ≤1, probing pocket depths ≤3, gingival index ≤1) and twenty-five never-smokers with generalized moderate to severe chronic periodontitis (attachment loss ≥5, probing pocket depths ≥5, gingival index >1 in 30% or more sites) were recruited following clinical and radiographic examination and informed consent obtained. Exclusion criteria for both groups included diabetes, HIV infection, use of immunosuppressant medications, bisphosphonates, or steroids, antibiotic therapy, or oral prophylactic procedures within the preceding 3 months, and fewer than 20 teeth in the dentition. Sample size was estimated using the HMP package in R^19^, based on an 80% power to detect an effect size of at least 0.20 using weighted UniFrac distances as the primary outcome variable, assuming a two-sided significance level of 0.05.

### Sample collection

From healthy subjects, samples were collected and pooled from 15 mesial sites on teeth with CAL ≤ 1 mm, PD ≤ 3 mm, GI ≤ 1 and no BOP (shallow-healthy) using sterile endodontic paper-points (Caulk-Dentsply, Milford, DE, USA). From the disease group, subgingival plaque from four nonadjacent proximal sites with attachment loss (CAL) ≥ 5 mm, probe depths (PD) ≥ 6 mm, bleeding on probing (BOP), and Loe and Silness gingival index (GI) ≥ 2 was collected using 15 paper points and pooled (deep-diseased). Samples were similarly acquired from four sites with CAL ≤ 1 mm, PD ≤ 3 mm, GI ≤ 1 and no BOP and separately pooled (shallow-diseased).

### DNA isolation and sequencing

Bacterial DNA was isolated from paper points, using Qiagen DNA MiniAmp kit (Qiagen, Valencia, CA, USA) and quantified using Qubit fluorometer. Library generation was completed using an Illumina TruSeq kit according to the manufacturer’s instructions. Briefly, genomic DNA was sheared enzymatically yielding an average fragment size of 500 base pairs. The fragment ends were blunted and adenylated, before ligation of barcodes and sequencing adaptors. Quantified and pooled libraries were clustered on the Illumina MiSeq (Illumina, Inc., San Diego, California), and 150 bp paired-end sequencing was performed in a commercial facility (Molecular Research LP, Shallowater, TX).

### Metagenomic Analysis

Trimmed and filtered sequences were uploaded to the MG-RAST metagenomics analysis pipeline (version 3.3.6)^20^,^21^ (Argonne National Laboratory) for quality processing and basic functional analysis. The MG-RAST API^22^, and the custom Python library we developed to access it and analyze/visualize results, were used throughout the analysis process to download relevant data and pipeline results (available for download at http://github.com/smdabdoub/PyMGRAST). Comparisons of functional potential between clinical groups were made in the context of the KEGG (Kyoto Encyclopedia of Genes and Genomes)^23^ and the SEED^24^ ontological hierarchies and statistical analysis of differential functional potential was performed using R and DESeq2^25^. Taxonomic identities for archeal, fungal, and viral sequences were assigned using the Lowest Common Ancestor (LCA) alignment to the M5NR database^26^. Bacterial rRNA genes were filtered from the metagenome by BLAT^27^ search against 90% clustered SILVA^28^. Rarefaction curves were generated from the 16S data and used to estimate sequencing coverage (Supplementary Fig. 1A). Taxonomic profiles were generated by comparing these filtered rDNA sequences to the HOMD database^29^ using the QIIME (version 1.8) and PhyloToAST (version 1.2) pipelines as previously described^30^. A core microbiome was computed for health and disease to include all s-OTUs (core phylome) and functional genes (core metagenome) present in at least 80% of the subjects in a group.

Contributions of each species to community function were assessed by network analysis of KEGG-annotated bacterial genome sequences. Taxonomic identifiers were assigned to the genes by alignment to the full set of 1528 genomes in the Human Oral Microbiome Database (HOMD) using Bowtie 2 (version 2.2.5)^31^. Sequences with multiple equivalent matches (within 10% alignment score) were assigned to the lowest common ancestor (LCA). The LSU genes of fungal sequences were similarly isolated, and identified by comparison to the SILVA database housed within MG-RAST. Species assignment was made at 97% identity^32^. Function-taxonomy networks were visualized using force directed networks (Fruchterman-Reingold^33^) and Force Atlas 3D^34^. Network-wide degree of specialization (H_2_′) was determined as a standardized degree of entropy, with 0.0 representing extreme generalization and 1.0 extreme specialization^35^,^36^. Specialization was calculated using version 2.05 of the R package bipartite^37^. The ability of genes to discriminate between health and disease was examined using a machine-learning algorithm (RandomForest package in R). The robustness of the classifier was evaluated using ROC curves (ROCR package in R). Two-thirds of the dataset was used to train the algorithm, which was tested on the remaining data. This was iterated 10 times and the mean ‘importance’ computed for each marker gene. The importance classification was used to select marker genes based on the methodology of Diaz-Uriarte^38^. For each iteration of the test, a ‘confusion table’ was created for each of the marker genes based on the number of correctly classified and misclassified samples; and this data was used to compute sensitivity and specificity.

## Results

An average of 3.32 million sequences per sample (range 1.97–4.53 million) were obtained for the shallow-healthy, shallow-diseased, and deep-diseased groups respectively. On average 77.22% of the shallow-healthy, 70.16% of the deep-diseased and 62.96% of the shallow-diseased sequences were human sequences. This is line with other investigations using similar approaches to study the subgingival microbiome^13^,^39^,^40^. Average coverage per sample ranged from 38% to 93% based on Nonpareil^41^, and was not significantly different between groups (p > 0.05, Tukey HSD, Supplementary Figure 1B). These sequences represented 4837 functionally annotated genes of bacterial, fungal, viral and archeal origin. The distribution of the sequences in each sample is shown in Supplementary Figure 2. Overall, 95% or more of the sequences belonged to bacteria. Viruses, archeae and fungi were identified in the samples, but were variably distributed. An average of 7% (range from 2.5–9.3%) of the sequences encoded for rRNA.

### Energy efficiency and functional equitability are central characteristics of a health-compatible microbial ecosystem

3348 functions were identified from periodontally healthy subjects, of which 1811 were present in the core healthy metagenome (≥80% of the healthy group); suggesting that in health, more than half of the microbial metagenome is conserved among all individuals.

**Figure 1:**
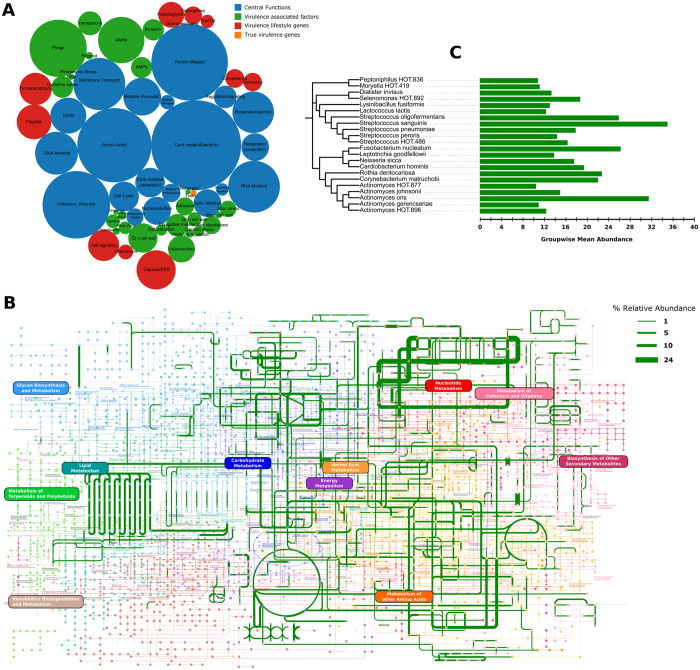
Predominant functionalities in health. (**A**) Shows a circle-packing graph of core genes grouped into higher order functions. Circles are sized by relative abundances of genes contributing to each function. (**B**) Shows a KEGG map of the core metabolic pathways in the health-associated microbiome. The lines are sized by log fold abundances. The genes and pathways used to create this map are presented in Supplementary Table 2. (**C**) Shows a selected group of species that contributed to these functions. The species shown here belonged to the core microbiome (80% or more of healthy individuals). The green bars represent the relative abundances of the species in all samples.

The most abundant functional group represented metabolic potential. Within this group 29% of genes encoded for protein metabolism, with genes responsible for protein, amino acid, RNA and DNA biosynthesis predominating this group (Fig. 1A,B and Supplementary Table 2). The second most abundant functionality was carbohydrate metabolism (22%). Within this framework, the primary pathways were related to aerobic metabolism of monosaccharides, organic acids, polysaccharides and one-carbon sugars, with an abundance of co-factors as catalysts (Fig. 1B). 17% of the core genes contributed to oxidative phosphorylation (aminotransferases, TCA cycle, pentose phosphate shunt, electron transport (cytochrome and ubiquinone families) and membrane transport (Fig. 1B and Supplementary Table 2)).

The second most abundant group of functions encoded in health (28%) was related to virulence lifestyle, and included antimicrobial resistance genes (AMRs), cell-signaling, competence, peptidoglycan biosynthesis and non-siderophore type iron transport (Fig. 1B and Supplementary Table 2). Additionally, a large number of phages, transposons, and gram-negative cell wall components (ranging from 1.3% to 5.7% of the metagenome) were also observed in health.

75% of the community membership was made up of 46 species belonging to the genera *Streptococcus, Veillonella, Actinomyces, Corynebacterium, Neisseria, Fusobacterium* and *Selenomonas*, of which 22 belonged to the core (Fig. 1C). There were no viruses or archaebacteria in the core; however, the fungal species *Candida albicans* was identified in 87% of healthy individuals. Co-occurrence networks demonstrated strong correlations (Spearman’s ρ > 0.8, p < 0.05) between *Actinomyces gerencseriae, A. oris, A. johnsonii, Selenomonas noxia, S. sputigena, S. artemides, Streptococcus sanguinis, S. oralis, Fusobacterium nucleatum* and *Veillonella parvula*. Each of the species contributed 879 ± 137 genes. While there was 60% overlap in the genes that each species contributed, there was a 98% overlap in the functions encoded by these genes, indicating that these species contribute to similar functions in the health-compatible microbiome (functional equitability or functional generalization). Some level of functional specialization was observed, with 6 species contributing genes encoding for motility and chemotaxis. Together, these 6 species (*Campylobacter rectus, C. showae, C. curvus, Selenomonas noxia, Eubacterium yurii*, and *Centipeda periodontii*) formed 0.019% of the core microbiome, and hence, were rare taxa within the microbiome.

### Global dysbiosis in disease

In addition to collecting samples from sites with clinical disease (deep-diseased), samples were collected from clinically healthy sites in subjects with disease (shallow-diseased) and from healthy subjects (shallow-healthy). The shallow-diseased sites were clinically similar to the shallow-healthy sites (Supplementary Table 1). If periodontitis were a result of a site-specific dysbiosis, then we would expect to see marked differences between deep-diseased and shallow-diseased samples, as well as significant similarities between shallow-healthy and shallow-diseased samples.

**Figure 2:**
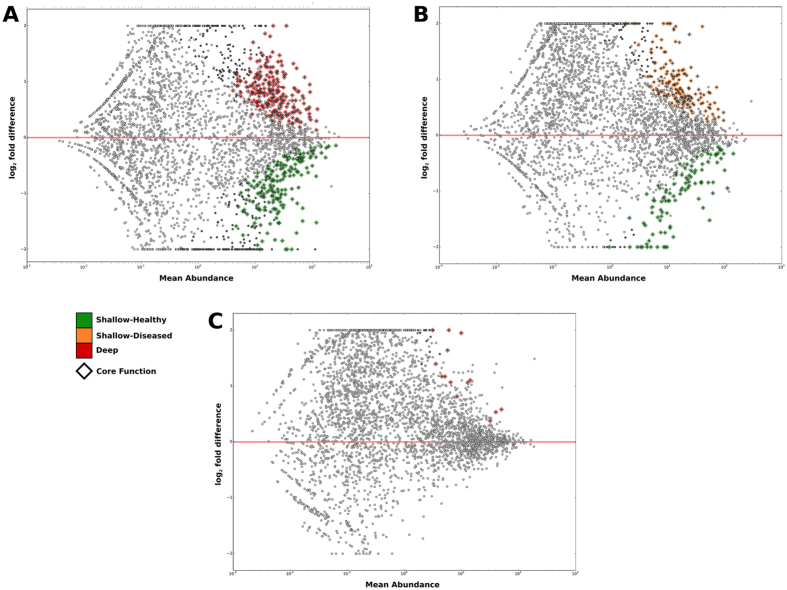
Bland-Altman plots of metagenomic differences between healthy, shallow and deep sites. Relative mean abundances of genes were plotted against log differences in abundance between groups. (**A**) Shows differences between healthy subjects and deep sites in subjects with periodontitis. Genes that were significantly overrepresented in deep sites (p < 0.05, FDR adjusted Wald test) are in red, those whose levels were significantly greater in health are in green. The central red line represents a log fold difference of zero. (**B**) Shows differential abundances between shallow sites (in orange) in subjects with periodontitis and healthy subjects (in green), while comparisons between shallow and deep sites in subjects with periodontitis are shown in (**C**). The genes and functions that were used to create these plots are shown in Supplementary Table 3.

Relative abundances of functional genes and taxa in the three groups were compared using DESeq2^25^. Shallow-healthy and deep-diseased sites demonstrated significant differences in nearly 2000 genes (p < 0.05, FDR adjusted Wald test), 1730 of which were part of the core metagenomes (Fig. 2A). Shallow-healthy and shallow-diseased sites differed in 1000 gene abundances (Fig. 2B), again, 880 of these belonged to the core. Few differences were detected between shallow-diseased and deep-diseased sites (Fig. 2C).

**Figure 3:**
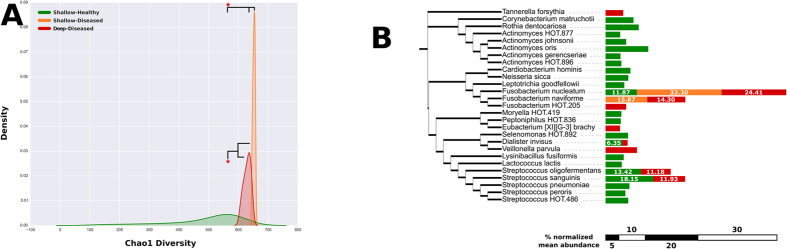
Alpha and beta phylogenetic diversity in health and disease. Kernel plots of Chao diversity index of healthy subjects and shallow and deep sites of subjects with periodontitis are shown in (**A**). Significant differences (p < 0.05, Tukey HSD) are indicated by an asterisk (*). Taxonomic differences between core microbiomes of health and disease are shown in (**B**). The bars represent the mean relative abundances of each species in each group.

**Figure 4:**
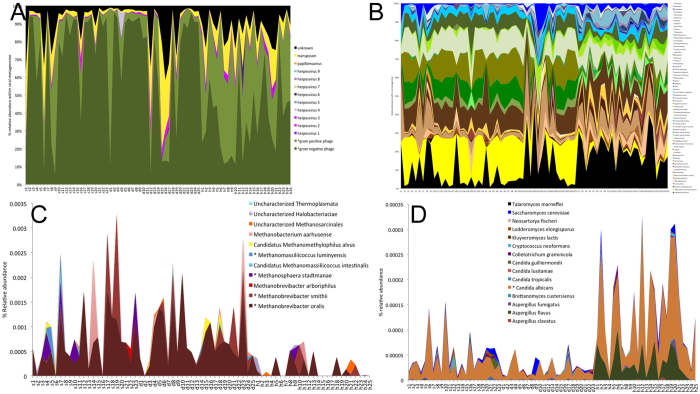
Non-bacterial members of the metagenomes of periodontal health and disease. Distribution of viral species by sample is shown in (**A**) and viral genes in (**B**). Relative abundances of species belonging to the archaebacterial kingdom in each sample is shown in (**C**,**D**) shows the distribution of species belonging to the fungal kingdom by sample. Genes and taxa that were significantly different between deep-diseased and shallow-healthy sites (p < 0.05, FDR adjusted Wald test) are indicated by an asterisk (*) in the legend.

Phylogenetically, both deep-diseased and shallow-diseased sites demonstrated a significantly higher alpha diversity (Chao) when compared to shallow-healthy sites (p < 0.05, Tukey HSD, Fig. 3A). Higher levels of several species (notably those belonging to the genera *Porphyromonas, Fusobacterium, Fretibacterium, Filifactor, Parvimonas, Selenomonas, Treponema* and *Kingella*), and lower levels of health-compatible species were observed in disease (p < 0.05, FDR adjusted Wald test, Fig. 3B and Supplementary Table 4). Shallow-diseased sites also exhibited lower levels of *Dialister invisus, Tannerella forsythia, Fusobacterium nucleatum* and Fretibacterium HOT.452 when compared to deep-diseased sites. There were no significant differences in the abundances of human viral species between groups (Fig. 4A). In shallow-healthy sites, gram-positive phages, especially those belonging to the genera *Streptococcus, Enterococcus* and *Lactobacillus* predominated (p < 0.05, FDR adjusted Wald test), while in disease (both shallow-diseased and deep-diseased sites) gram-negative phages associated with *Prevotella, Burkholderia, Campylobacter, Haemophilus* and *Aggregatibacter* were significantly more abundant (Fig. 4A and B). The abundances of the archebacterial species *Methanobrevibacter oralis, M. smithii, Methanomassiliicocus luminyensis* and *Methanosphaera stadtmaniae* were significantly higher in disease when compared to health (Fig. 4C). Shallow-diseased and shallow-healthy sites demonstrated higher levels of *Candida albicans* when compared to deep-diseased sites (Fig. 4D).

### Expanded functional capabilities and functional specialization in disease

**Figure 5:**
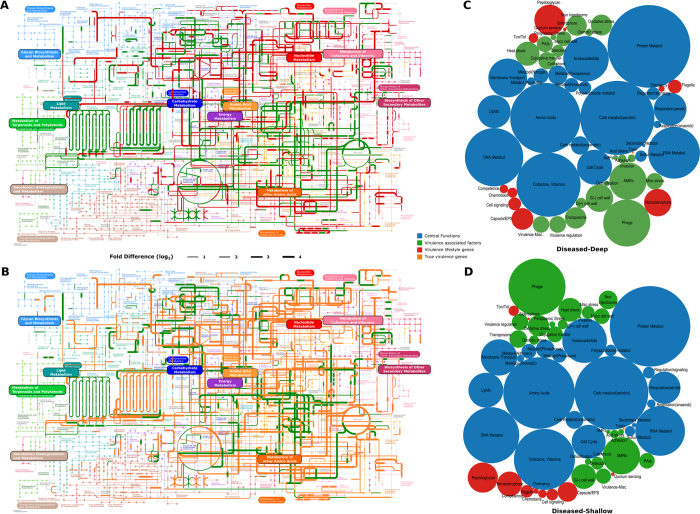
Metabolic differences between health and disease. KEGG maps of differences between healthy subjects and deep sites are shown in (**A**). The pathways are sized by relative abundances (Log scale) of genes contributing to the functionality. (**B**) shows a circle-packing graph of core genes in deep-diseased sites grouped into higher order functions. Circles are sized by relative abundances of genes contributing to each function Differences between healthy subjects and shallow sites are shown in (**C**) and core genes in shallow-diseased sites in (**D**). The genes and functions that were used to create these maps are shown in Supplementary Table 3.

Out of the 4837 functional units identified in the present investigation, 2612 were common to both health and disease, 1489 were uniquely observed in disease (of which 1249 belonged to the disease-associated core microbiome) and 736 uniquely in health (657 in the health-compatible core microbiome). The common genes predominantly encoded for central functions such as carbohydrate and protein metabolism, aerobic respiration, protein and amino acid synthesis and virulence. However, the abundances of these common genes were significantly different between groups (p < 0.05, FDR adjusted Wald test, Fig. 2A and B, and Supplementary Table 3). The genes unique to disease encoded for fermentation, antibiotic resistance, detoxification stress, adhesion, invasion and intracellular resistance, proteolysis, quorum sensing, Type III and IV secretion systems, phages and toxins and superantigens (Fig. 5 and Supplementary Table 3). Significant over-representation (p < 0.05, FDR adjusted Wald test) of genes associated with oxidation of primary alcohols, butyrate, isovalerate, propionate, acetate, glycolate and aromatic compounds was observed in disease (Fig. 5, and Supplementary Table 3). Furthermore, while shallow-healthy samples demonstrated high levels of aerobic reductases, anaerobic reductases were almost exclusively found in disease, and constituted 1.2% of this metagenome. Strong and significant correlations (r^2^ > 0.7, p < 0.05) were observed between bacterial fermentation and archeal methanogenesis genes (especially coenzyme F420, coenzyme B, coenzyme M, methanofuran, and methanopterin) in shallow-diseased and deep-diseased but not shallow-healthy sites (Supplementary Figure 3). Very few sulfate-reducing genes were identified in disease.

49 genes contributing to flagellar motility and 14 genes encoding for chemotaxis were identified in the core disease microbiome. Of these, 28 genes, especially those contributing to assembly of the filament, hook, basal body, rods and rings (FlhA/B, FlgG/C, FliG/I/J/K/L/S, FlgC/G) were either over-represented or uniquely represented in deep-diseased and shallow-diseased sites when compared to shallow-healthy samples (p < 0.05, FDR adjusted Wald test, Fig. 5, and Supplementary Table 3). Type III secretion systems (FlhA/B and FliI) were also significantly higher in disease when compared to health. Flagellar proteins play important roles not only in bacterial motility, but also in adhesion, Type III secretion and virulence. Flagellins belong to the family of PAMPS and stimulate innate and adaptive immune responses through TLR5^42^.

Overall, 28% of the genes in the shallow-healthy, 36.8% in shallow-diseased and 38.2% in deep-diseased sites encoded for virulence. However, in health, these genes accounted for 8.9% of the genome abundance, while in disease, 33.1% of the genomic content was attributable to virulence. The greatest differences between health and disease were observed in antibiotic resistance, iron acquisition, and gram negative and gram-positive cell wall components (p < 0.05, FDR adjusted Wald test, Fig. 5 and Supplementary Table 3). The abundances of efflux pumps ranged from 0.07 to 0.6% of the genomic content, and a statistically significant correlation (r^2^ = 0.61, p = 0.0036) was observed between efflux pumps and gram-negative phages in disease, but not in health (Supplementary Figure 4C). EPs are a three-component system (comprising an outer membrane protein, a periplasmic fusion protein and an inner membrane protein), which displace toxic compounds (including antibiotics) from gram-negative bacteria. Although EPs are an intrinsic part of the genome of several gram-negative bacteria, within an ecosystem these genes are typically acquired either through horizontal gene transfer or through mutations^43^,^44^. A similar correlation was also observed between LPS, metal resistance and efflux pumps (Supplementary Figure 4A and B). LPS is most known for being a powerful antigen that triggers a florid inflammatory response, however, its role in the bacterium is one of barrier function. Our data corroborate previous studies in the literature suggesting a synergy between cell exclusion and efflux in mediating antibiotic resistance^45^.

Iron is important for bacterial survival since it facilitates electron transport, nucleotide synthesis, peroxide reduction and other essential cellular functions. The host typically sequesters iron by complexing it as hemoglobin, or by storage proteins such as ferritin and lactoferrin (nutritional immunity). Bacteria take up iron either directly from heme and heme-containing compounds using surface receptors and ABC transporters, or indirectly using high affinity small-molecule chelators known as siderophores. Bacteria also use iron availability as a metric to sense their environment; iron-deprivation leads to expression of several outer membrane proteins, siderophores, hemolysins and toxins, while availability of iron promotes pathogen expansion and cellular invasion. Iron transport genes accounted for 1.3% of the health-associated genome and 5% of the disease-associated metagenome. Both siderophoric and non-siderophoric transport mechanisms were significantly different between health and disease.

Lipid-A, the lipid moiety of LPS, is a powerful antigen that elicits a florid pro-inflammatory host response. Lipid-A synthesis is known to be upregulated in the presence of hemin^46^. Genes responsible for Lipid-A synthesis were preferentially enriched in disease when compared to health, with *Capnocytophaga, Campylobacter, Fusobacterium, Porphyromonas, Prevotella, Tannerella*, and *Treponema*, as major contributors. The combined enrichment of both iron acquisition genes and Lipid A in disease suggests that this microbiome has the potential for a virulent transcriptional profile in the presence of blood (i.e. in conditions of inflammation, such as gingivitis).

Genes responsible for management of oxidative stress formed 4.12% of shallow-healthy sites and 0.98% of disease (p < 0.05, FDR adjusted Wald test, Fig. 5, and Supplementary Table 3). The disease-associated microbiome, on the other hand, demonstrated a greater abundance of rubrerythrin and sigma factors. Glutathione is an important redox-buffering compound that protects bacterial cells from osmotic stress, electrophiles and oxidative stress and by acting as an electron donor during reduction of lipid peroxides and hydroperoxides, and for scavenging reactive oxygen. The present investigation suggests that the health-compatible microbiome is well equipped to handle oxidative stresses through the glutathione, and that this functionality is not as marked in disease. Rubrerythrin is a non-haem iron compound that protects anaerobic bacteria such as *P. gingivalis* from reactive oxygen and nitrogen species, both of which are produced during a neutrophil-mediated host response^47^. This mechanism enables growth and tissue invasion by the organism. Sigma factors are dissociable subunits of RNA polymerase. Recent evidence indicates that these factors may play a major role in enabling bacterial transition from a free-living state to host invasion^48^ by regulating the expression of several virulence genes. Thus, the data indicate that the disease-associated microbiome possesses the capabilities for host tissue invasion in response to environmental stress.

**Figure 6:**
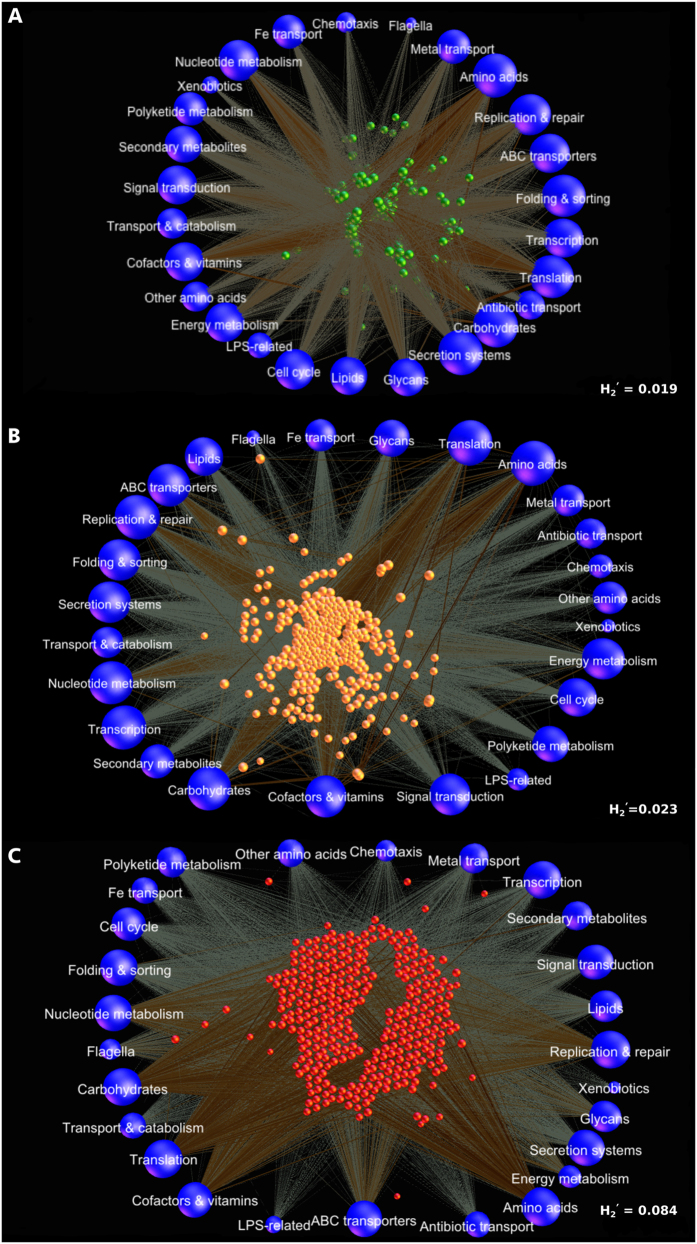
Functional contributions of bacterial species in the subgingival metagenome. Force-directed networks of bacterial species and their contribution to metabolic pathways in health (**A**), shallow sites (**B**) and deep sites (**C**). Each network graph contains nodes (circles) and edges (lines). Nodes in the center of each network represent species-level OTU’s in healthy (green), deep sites (red) and shallow sites (orange) and nodes on the outer edge represent the functional contributions of these species. Edges represent the number of genes contributed by each species to each functional family. Only significant correlations between species and their functional contributions (p < 0.05, t-test) with a coefficient of at least 0.75 are shown. The data used to create these networks are presented in Supplementary Table 5. Few species-level nodes can be seen in health, with equal number of edges connecting each of these species to the functional nodes. Both deep and shallow sites demonstrate larger numbers of species-level nodes than health. Moreover, while many functions species are connected to their cognate species by large numbers of edges, certain functions have contributions only from a few species. This is numerically indicated by the degree of functional specialization (H2′).

A significantly higher degree of functional specialization was evident in disease when compared to health (p < 0.05, Tukey HSD of H_2_′, Fig. 6). 547 bacterial, viral, and archebacterial s-OTUs were identified in disease (148 ± 23 in each sample). Each species contributed an average of 867 genes, however the range varied from 97 genes (Archebacteria, *Treponema, Synergistes*, TM5) to 1256 genes (*Streptococcus, Neisseria, Actinomyces, Filifactor, Dialister, Porphyromonas, Fusobacterium, Eubacterium*). Also, while more than 400 species contributed genes encoding for respiration, protein and carbohydrate metabolism, 32 species contributed flagellar genes, 69 contributed genes for glycan synthesis and 76 species encoded for LPS.

### Taxonomically idiosyncratic yet functionally congruent communities in disease

Of the 547 s-OTUs identified in disease, only 9 were found in the core microbiome associated with disease (Fig. 3B). On the other hand, the core metagenome of deep-diseased sites comprised of 1207 out of 3855 functional units, while that of shallow-diseased sites comprised 1211 out of 4137 functional units, indicating that one third of the functionality is conserved among all sites in an individual with disease.

**Figure 7:**
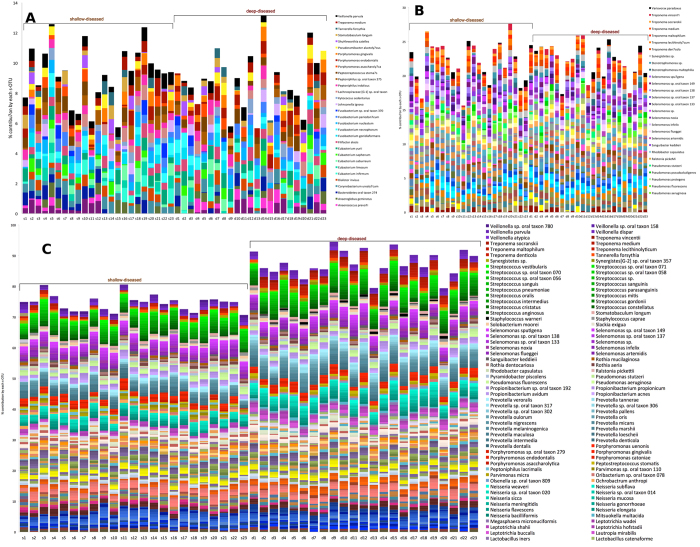
Phylogenetic distribution of functional potential in health and disease. Distribution of taxa encoding for fermentation (**A**), flagella (**B**) and iron acquisition (**C**) by sample. 23 paired samples of shallow and deep sites in subjects with periodontitis and 25 samples from periodontally healthy subjects are shown.

The predominant bacterial species responsible for fermentative pathways were *Anaerococcus lactolyticus, A. prevotii, Anaeroglobus geminatus*, Bacteroidetes oral taxon 274, *Corynebacterium urealyticum, Dialister invisus, Eubacterium infirmum, E. limosum, E. saburreum, E. saphenum, E. yurii, Filifactor alocis, Fusobacterium gonidiaformans, F. necrophorum, F. nucleatum, F. periodonticum*, Fusobacterium sp. oral taxon 370, *Johnsonella ignava, Kytococcus sedentarius*, Lachnospiraceae [G-1] sp. oral taxon, *Peptoniphilus indolicus*, Peptoniphilus sp. oral taxon 375, *Peptostreptococcus stomatis, Porphyromonas asaccharolytica, P. endodontalis, P. gingivalis, Pseudoramibacter alactolyticus, Shuttleworthia satelles, Stomatobaculum longum, Tannerella forsythia, Treponema medium*, and *Veillonella parvula*. However, different bacterial consortia contributed to fermentation in each subject, even between shallow and deep sites within each subject (Fig. 7A). Similarly, although flagellar genes formed part of the core microbiome of disease, several different species contributed to these functions in each sample. For example, in certain individuals, the Treponemes were the dominant contributors of flagellar function, while in certain others it was the Selenomonads or Campylobacters (Fig. 7B). Although there were no statistically significant differences in the abundances of iron acquisition genes between shallow-diseased and deep-diseased sites, in shallow-diseased sites, this was attributable to species belonging to the genera *Neisseria, Bifidobacterium, Porphyromonas, Selenomonas, Actinomyces* and *Streptococcus*, while in deep-diseased sites of the same individuals *Prevotella, Lactobacillus, Fusobacterium* and *Treponema* contributed to a large fraction of this functionality (Fig. 7C).

All three red complex bacteria (*Porphyromonas gingivalis, Treponema denticola* and *Tannerella forsythia*) were detected in 23 out of the 73 samples. At least one of these species was detected in 45 samples. Principal co-ordinate analysis of the functional genes did not reveal significant clustering of the samples based on presence or absence of these species. Moreover, a Random Forest machine-learning algorithm did not identify any species or consortia in the core microbiomes of health or disease that could discriminate between health and disease. However, the algorithm did identify 31 functional genes within the core microbiomes of health and disease that were capable of discriminating between health and disease. Together, this panel of genes was capable of classifying subjects into the health and disease categories with 99% sensitivity and 100% specificity (Table 1).

## Discussion

There have been several investigations in recent years that have examined the functional potential of the subgingival microbiome in health and disease^12^,^13^,^14^,^40^,^49^. While some of these investigations have used a targeted DNA-array based approach to examine selected functions^14^, others have been limited by small sample sizes in making statistical comparisons^12^,^13^,^40^,^49^. Moreover, all of these studies have focused only on the bacteriome. Since there is considerable evidence in the literature that viruses, archaea and fungi are common inhabitants of the subgingival microbial ecosystem and may play a role in health or disease^17^,^18^,^50^, we examined the metagenomes of all these domains using a comprehensive open-ended approach on a sample size large enough to permit robust statistical inferences to be made. There is little evidence in the literature on what fraction of the subgingival microbiome is comprised of viruses and fungi. In the present investigation, only 5% of the genomic abundance was attributable to these taxonomic clades, since we did not specifically enrich for these organisms. While it is possible that these taxa were underrepresented in the present investigation, previous investigations have reported that the proportions of viruses in complex microbial communities can be accurately estimated without enrichment strategies^51^. Moreover, other investigations have reported similar proportions of these organisms^13^.

Since the oral cavity is an open microbial ecosystem with transient members (allochthonous constituents) and stable colonizers (autochthonous community), we examined the core metagenome^52^ of health and disease to minimize the effect of allochthonous species and genes on the analysis. We defined the core metagenome as that which is found in 80% or more of individuals. This is a far more conservative definition than that used by previous investigations^10^,^52^, and ensured that the genes under investigation were truly representative of the subgingival metagenome.

The term ecosystem describes a community of living organisms interacting as a system and linked to each other through energy transfer and nutritional flow^53^. Our data suggest that the health-compatible microbiome is a highly energy efficient ecosystem, with 68% of the genome dedicated to energy acquisition, transfer and utilization. The genomic framework is set up for energy acquisition mainly through carbohydrate metabolism. Energy transfer is mediated mainly through oxidative phosphorylation; the high levels of aminotransferases and the robust glutamate pathway point to the citric acid cycle as a preponderant energy transfer mechanism. This is possibly facilitated by high oxygen tensions that prevail in the healthy gingival sulcus^54^. Strong co-occurrence patterns among known nutritional and structural symbionts (e.g., Veillonella, Streptococci, Actinomyces and Fusobacteria) attest to functional cooperativity in this system.

On the other hand, in periodontitis (both shallow and deep sites), fermentation and methanogenesis are the predominant pathways for energy acquisition. Effective fermentation requires the presence of a ‘hydrogen sink’, that is, sulfate-reducing or methanogenic species^55^. The robust archeal presence, sparsity of sulfate-reducing genes and correlations between abundances of bacterial fermentation genes and archeal methanogenesis genes suggest syntrophic interspecies hydrogen transfer between archaebacteria and eubacteria in periodontal disease, which corroborates previous hypotheses that the presence of archaea may promote colonization by fermenters^17^,^56^. Fermentation of one mole of glucose yields 2–4 molecules of ATP when compared to aerobic respiration, which yields 32–36 ATP. Also, the end products of fermentation, short chain fatty acids such as butyrate, propionate and isobutyrate for example, have been strongly associated with periodontitis^57^,^58^. Thus, our data suggest that the disease-associated microbiome lacks the capability for efficient energy processing, forcing this community to do ‘hard work’ rather than ‘smart work’ for survival, and that the by-products thus created may contribute to disease etiology.

Recent paradigms of disease pathogenesis have promulgated the ‘pathobiont’ hypothesis, which states that disease occurs due to expansion of certain members of the indigenous microbiome rather than acquisition of new species^59^,^60^. To investigate this, the transition from health to disease was modeled by comparing the core microbiomes of healthy subjects to sites with and without clinical disease in subjects with disease. Phylogenetically and functionally, diversity progressively increased from shallow-healthy to shallow-diseased to deep-diseased sites; and this was due to both increase in abundances of certain indigenous members and functions as well as addition of new members and their associated functions. Importantly, a central feature of health was that all microbial members contributed genes that perform the functions required by this ecosystem. Thus, the health-compatible ecosystem appears to be a generalist microbiome. By contrast, disease is dominated by ‘specialist organisms’, which encode for novel metabolic functions (e.g., proteolysis, fermentation, methanogenesis) or virulence factors (e.g., motility, communication, stress response, iron acquisition, antibiotic resistance) not seen in health. Furthermore, functional cooperativity between bacteria, viruses and archaea is more readily observed in disease than in health and, while a strong core microbiome was detected in health, it was conspicuously absent in disease. Taken together, the microbial heterogeneity, the predominance of specialist species and the presence of novel functions that correlate with the requirements of the environment suggest that many more microbial events underlie the etiology of periodontitis than simple pathobiont expansion.

It has previously been shown that a gradient exists in the levels of pro-inflammatory cytokines, oxygen tension and antioxidant capacity between shallow-healthy, shallow-diseased and deep-diseased sites^6^,^61^. Our data suggest that a progressive regime shift occurs in the microbial ecosystem from health to disease, which is reflective of the gradients in the local microenvironment. This, of course, leads to the question whether the disease microbiome is the cause or the product of the disease. To answer this, we compared shallow-diseased sites with deep-diseased sites. The core microbiomes of shallow-diseased sites were phylogenetically distinct from, but functionally more aligned with deep-diseased than with shallow-healthy sites (especially in energy processing, virulence, chemotaxis, stress response and phage-mediated transfers), indicating that the microbiomes of these sites do indeed, have the potential to induce disease. This observation that shallow-disease sites possess similar pro-inflammatory abilities to disease active sites serves in part, to explain the random burst model of disease activity. However, since the potential to cause disease does not equate to disease causation, this needs to be corroborated by longitudinal investigations of the microbial metatranscriptome during shifts from health to disease.

Several decades of research have explored the possibility of identifying species that would serve as markers or predictors of disease^62^,^63^,^64^,^65^,^66^,^67^. However, the present investigation demonstrates that while a tremendous functional complementarity exists in disease, this microbiome is taxonomically heterogeneous. Further, 30 genes found in the core-disease microbiome (corresponding to 14 distinct functions) were capable of discriminating between health and disease. Also, these genes were present in both shallow-diseased and deep-diseased sites, indicating that any site may be sampled to screen subjects for disease. Thus, our data suggest that a gene-centric rather than a species-centric approach to identifying markers and predictors may be more fruitful.

## Conclusions

The central characteristics of the health-compatible subgingival microbial community are energy efficiency and functional equitability. Fewer functions are encoded within this healthy microbiome, and the general functional potential is distributed across most species, while expansion of functional capabilities can be observed in disease, within certain species or consortia contributing a few, unique functions. Disease is also characterized by taxonomic heterogeneity and functional congruence. Importantly, sites without clinical disease in subjects with disease are functionally more aligned with sites with disease than with healthy sites, indicating that they may be more at-risk-for-harm than previously believed.

## Additional Information

**How to cite this article**: Dabdoub, S. M. *et al*. Comparative metagenomics reveals taxonomically idiosyncratic yet functionally congruent communities in periodontitis. *Sci. Rep.* **6**, 38993; doi: 10.1038/srep38993 (2016).

**Publisher's note:** Springer Nature remains neutral with regard to jurisdictional claims in published maps and institutional affiliations.

## Supplementary Material

Supplementary Dataset 1

Supplementary Dataset 2

Supplementary Dataset 3

Supplementary Dataset 4

Supplementary Dataset 5

## Figures and Tables

**Table 1:** Candidate marker genes.

Gene	Functional role	Association
N-methylhydantoinase (ATP-hydrolyzing) (EC 3.5.2.14)	Amino acid derivatives	Disease
Putrescine transport ATP-binding protein PotG (TC 3.A.1.11.2)	Amino acid derivatives	Disease
Heterodisulfide reductase	Anaerobic respiratory reductases	Disease
Deoxyribonuclease YjjV	Carbohydrate Metabolism	Health
Dihydrolipoamide dehydrogenase (EC 1.8.1.4)	Carbohydrate Metabolism	Health
Hydroxypyruvate reductase (EC 1.1.1.81)	Carbohydrate Metabolism	Health
Iron-containing alcohol dehydrogenase	Carbohydrate Metabolism	Health
Pyruvate oxidase (EC 1.2.3.3)	Carbohydrate Metabolism	Health
Chromosome (plasmid) partitioning protein ParB-2	Cell Division	Health
Flavodoxin 2	Cofactors, Vitamins, Prosthetic Groups, Pigments	Disease
CRISPR-associated RAMP Cmr4	CRISPs	Disease
CRISPR-associated RecB family exonuclease Cas4b	CRISPs	Disease
Stage V sporulation protein	Dormancy and Sporulation	Disease
UDP-2,3-diacylglucosamine hydrolase (EC 3.6.1.−)	Gram-Negative cell wall components	Disease
N-acetylmannosaminyltransferase (EC 2.4.1.187)	Gram-Positive cell wall components	Disease
Haemin uptake system permease protein	Iron Acquisition	Disease
Membrane fusion protein (MFP) component of efflux pump	Membrane Transport	Disease
Na(+) H(+) antiporter subunit A	Membrane Transport	Disease
Nudix hydrolase	Phage regulation of gene expression	Disease
Co/Zn/Cd efflux system membrane fusion protein	Resistance to antibiotics and toxic compounds	Disease
Cobalt-zinc-cadmium resistance protein CzcD	Resistance to antibiotics and toxic compounds	Disease
rRNA adenine N-6-methyltransferase (EC 2.1.1.48)	Resistance to antibiotics and toxic compounds	Disease
BatC (Bacteroides aerotolerance operon)	Respiration	Disease
Cytochrome c oxidase polypeptide I (EC 1.9.3.1)	Respiration	Disease
Cold shock protein CspC	Stress Response	Health
Ferric siderophore transport system, biopolymer transport protein ExbB	Ton and Tol transport systems	Disease
Flagellar biosynthesis protein FliQ	Virulence, Disease and Defense	Disease
Flagellar biosynthesis protein FliS	Virulence, Disease and Defense	Disease
Flagellar motor rotation protein MotB	Virulence, Disease and Defense	Disease
Hemolysin III	Virulence, Disease and Defense	Disease
Inner membrane protein CreD	Virulence, Disease and Defense	Disease

The ability of genes to discriminate between health and disease. The genes that were identified using random forest and their predicted function are shown.
